# A codon substitution model that incorporates the effect of the GC contents, the gene density and the density of CpG islands of human chromosomes

**DOI:** 10.1186/1471-2164-12-397

**Published:** 2011-08-06

**Authors:** Kazuharu Misawa

**Affiliations:** 1 Research Program for Computational Science, Research and Development Group for Next-Generation Integrated Living Matter Simulation, Fusion of Data and Analysis Research and Development Team, 1-7-22 Suehiro-cho, Tsurumi-ku, Yokohama City, Kanagawa, 230-0045, Japan

**Keywords:** Rate of molecular evolution, CpG hypermutability, codon substitution, gene density, chromosomal GC content

## Abstract

**Background:**

Developing a model for codon substitutions is essential for the analyses of protein sequences. Recent studies on the mutation rates in the non-coding regions have shown that CpG mutation rates in the human genome are negatively correlated to the local GC content and to the densities of functional elements. This study aimed at understanding the effect of genomic features, namely, GC content, gene density, and frequency of CpG islands, on the rates of codon substitution in human chromosomes.

**Results:**

Codon substitution rates of CpG to TpG mutations, TpG to CpG mutations, and non-CpG transitions and transversions in humans were estimated by comparing the coding regions of thousands of human and chimpanzee genes and inferring their ancestral sequences by using macaque genes as the outgroup. Since the genomic features are depending on each other, partial regression coefficients of these features were obtained.

**Conclusion:**

The substitution rates of codons depend on gene densities of the chromosomes. Transcription-associated mutation is one such pressure. On the basis of these results, a model of codon substitutions that incorporates the effect of genomic features on codon substitution in human chromosomes was developed.

## Background

Accurate models of DNA and protein evolution are essential for studying molecular evolution. Models of DNA and protein evolution are used in homology searches [1], sequence alignments [2,3], gene finding [4], detection of natural selection [5-8], and reconstruction of phylogenetic trees [9,10].

Recent studies on the mutation rates in non-coding regions have shown that the mutation rates of CpGs in the human genome are negatively correlated to the local GC content [11-14] and to the densities of functional elements [11].

CpG hypermutability [15] is known to be one of the major causes of codon substitution in mammalian genes [16-19]. CpG dinucleotides are often methylated at C, and methylated C spontaneously deaminates to thymine (T) approximately 10 times [20] or more [21] rapidly than other types of point mutations. Approximately 14% of codon substitutions are caused by CpG hypermutations [22]. Statistical indications of positive selection in some sequences or individual codons may be caused by a difference in mutation rates in the synonymous and nonsynonymous sites because of CpG hypermutations [23]. Previous studies that focused on the effect of CpG hypermutation on the rate of amino acid change in the human lineage [22,24] did not incorporate the regional variation in the rates of codon substitution. Thus, the regional variation in codon substitutions, especially those related to CpG hypermutation, in the human genome is of interest.

To understand the effect of these genomic features such as GC content, gene density, and frequency of CpG islands on the rates of codon substitution, the rates of codon substitution were estimated by using the coding regions of thousands of human and chimpanzee genes and inferring their ancestral sequences by assuming macaque genes as the outgroup. Since the genomic features are interdependent, partial regression coefficients of these features were obtained. Multiple regression analyses to evaluate the effect of GC content, gene density, and CpG island density on the rates of CpG to TpG substitutions, TpG to CpG substitutions, and non-CpG transitions and transversions were conducted.

## Results

The number of genes on each chromosome is listed in Table 1. Table 1 also shows the GC content and the gene and CpG island density on human chromosomes.

**Table 1:** Summary of data

Chromosome	GC Content (%)	Gene Density^a^	CpG Island Density^a^	Chromosome Size (base pair)	No. of genes used in this study	No. of codons used in this study
1	41.74	8.30	33.69	249250621	1123	504737
2	40.24	5.39	28.03	243199373	708	349066
3	39.69	5.68	18.20	198022430	644	311774
4	38.25	4.39	19.13	191154276	442	222017
5	39.52	5.24	24.77	180915260	521	266213
6	40.33	6.23	28.13	171115067	623	260600
7	40.75	6.45	42.13	159138663	472	214102
8	40.18	5.39	32.47	146364022	359	158173
9	41.31	5.94	35.58	141213431	420	192744
10	41.58	5.99	40.98	135534747	431	194136
11	41.57	10.24	38.98	135006516	593	255208
12	40.81	8.10	35.40	133851895	567	258243
13	38.53	2.95	23.29	115169878	198	100596
14	40.89	6.18	28.97	107349540	360	168500
15	42.20	6.91	29.00	102531392	321	165732
16	44.79	10.58	74.48	90354753	435	196533
17	45.54	15.48	81.09	81195210	549	246160
18	39.79	3.89	31.89	78077248	192	87095
19	48.34	25.28	134.13	59128983	525	205475
20	44.13	9.04	56.61	63025520	319	130636
21	40.84	5.17	44.32	48129895	127	54345
22	47.99	8.77	73.68	51304566	199	84980
X	39.50	5.62	19.66	155270560	244	90162

^a ^per Mb

**Figure 1:**
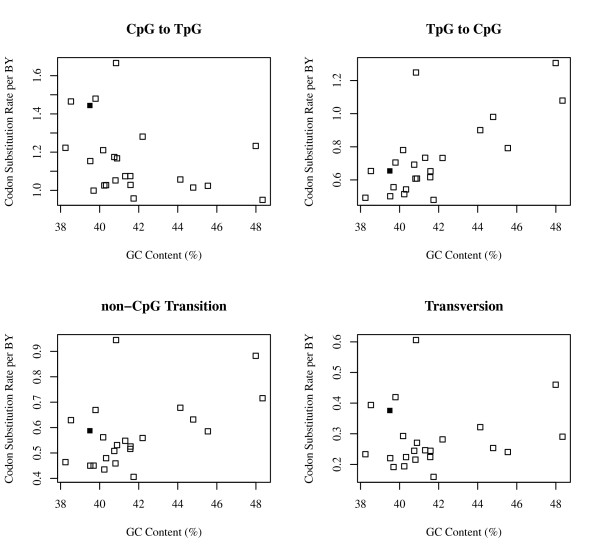
**Scatter plot of CpG and non-CpG substitution rates that are positively correlated to the number of CpG islands per site on the chromosome**. Open squares: Synonymous C to T substitution rate at CpG sites on the autosomes. Closed squares: Synonymous C to T substitution rate at CpG Sites on the X chromosome.

Figure 1 is a scatter plot of the synonymous rates of CpG to TpG and TpG to CpG substitutions, non-CpG transitions and transversions, and GC content of the chromosomes. The open squares (□) show the synonymous substitution rate on autosomes. The closed squares show the synonymous substitution rate on the X chromosome. In all the cases, substitution rates on the X chromosome are within the range of those on the autosomes.

Figure 1 also shows that the synonymous CpG to TpG substitution rates were negatively correlated to the GC content of the chromosomes. In contrast, TpG to CpG synonymous substitution rates were positively correlated to the GC content of the chromosomes. As seen in Table 2, these correlations were statistically significant (P < 0.001), and all types of substitutions have a significantly negative correlation to the gene density (P < 0.001).

**Table 2:** Correlation coefficient analysis between ln (substitution rate ×10^8^) and chromosomal features

Type	Grantham Distance	GC Content (%)	Gene Density^a^	CpG Island Density^a^	Chromosome Size
CpG to TpG	-0.23**	-0.04**	-0.03**	-0.08**	-0.13**
TpG to CpG	-0.39**	0.09**	0.09**	0.02*	-0.25**
non-CpG Transition	-0.35**	0.08**	0.08**	0.01**	-0.24**
Transversion	-0.23**	0.06	0.07**	-0.02**	-0.32**

*at 1% level of significance; **at 0.1% level of significance; ^a^per Mb

Since the genomic features are interdependent, partial regression coefficients of these features were obtained. Table 3 shows the rates of CpG to TpG and TpG to CpG substitutions and non-CpG transitions and transversions. Except TpG to CpG substitutions, all types of substitutions show a significant correlation with Grantham's distance, GC content, gene and CpG island density, and chromosome size (P < 0.001).

**Table 3:** Partial coefficient of Grantham distance, GC content, gene density, CpG island density, and chromosome size obtained by multiple regression analysis

Type	Partial Correlation Coefficient ± SD
Intercept	
CpG to TpG	5.13 ± 0.40
TpG to CpG	2.59 ± 0.04
non-CpG Transition	3.09 ± 0.17
Transversion	3.84 ± 0.14
Grantham Distance	
CpG to TpG	-5.05 × 10^-3 ^± 2.29 × 10^-4^**
TpG to CpG	-9.48 × 10^-3 ^± 2.25 × 10^4^**
non-CpG Transition	-7.81 × 10^-3 ^± 9.79 × 10^-5^**
Transversion	-3.91 × 10^-3 ^± 7.25 × 10^-5^**
GC Content (%)	
CpG to TpG	-7.13 × 10^-2 ^± 1.03 × 10^-2^**
TpG to CpG	-1.52 × 10^-2 ^± 1.01 × 10^-2^
non-CpG Transition	-3.23 × 10^-2 ^± 4.40 × 10^-3^**
Transversion	-6.18 × 10^-2 ^± 3.66 × 10^-3^**
Gene Density ^a^	
CpG to TpG	7.21 × 10^-3 ^± 1.68 × 10^-3^**
TpG to CpG	8.36 × 10^-3 ^± 1.62 × 10^-3^**
non-CpG Transition	8.14 × 10^-3 ^± 6.94 × 10^-4^**
Transversion	9.78 × 10^-3 ^± 5.55 × 10^-4^**
CpG Island Density ^a^	
CpG to TpG	-4.23 × 10^-2 ^± 6.72 × 10^-3^**
TpG to CpG	-5.38 × 10^-2 ^± 6.46 × 10^-3^**
non-CpG Transition	-4.64 × 10^-2 ^± 2.80 × 10^-3^**
Transversion	-5.76 × 10^-2 ^± 2.25 × 10^-3^**
Chromosome Size	
CpG to TpG	-3.83 × 10^-9 ^± 2.64 × 10^-10^**
TpG to CpG	-4.70 × 10^-9 ^± 2.52 × 10^-10^**
non-CpG Transition	-4.30 × 10^-9 ^± 1.08 × 10^-10^**
Transversion	-5.65 × 10^-9 ^^-9 ^± 8.40 × 10^-11 ^**

*at 1% level of significance; **at 0.1% level of significance; ^a^per Mb

Table 4 shows the difference between correlation coefficient squared (r^2^) obtained by single regression to Grantham distance and adjusted r^2 ^obtained by multiple regression to Grantham distance, GC content, gene density, CpG island density. This table shows that including GC content, gene density, CpG island density to the model increases r2 and improves the ability of prediction of the substitution rates.

**Table 4:** Correlation coefficients squared (r^2^) between ln (Rate ×10^8^) and chromosomal features

Type	r^2 ^obtained by single regression to Grantham distance	Adjusted r^2 ^obtained by multiple regression to Grantham distance, GC content, gene density, CpG island density
CpG to TpG	0.051	0.089
TpG to CpG	0.154	0.224
non-CpG Transition	0.122	0.182
Transversion	0.054	0.190

On the basis of these results, a model that incorporates these genetic features was developed. Parameter *x *is calculated as the sum of the intercept and the partial correlation coefficient multiplied by the genetic features, as shown in Table 1. For example, *x *for a synonymous CpG to TpG substitution on chromosome 1 is calculated as follows: 5.13 + (5.13 × 0) - (7.13 × 10^-2 ^× 41.74) + (7.21 × 10^-3 ^× 8.30 - 4.23 × 10^-2^) × (33.69 - 3.83 × 10^-9^) × 249250621. The codon substitution rate can be further calculated as exp(*x*).

## Discussion

In this study, codon substitution rates of CpG to TpG mutations, TpG to CpG mutations, and non-CpG transitions and transversions were estimated by comparing the coding regions of thousands of human and chimpanzee genes from entire genome and inferring their ancestral sequences by assuming macaque genes as the outgroup.

As seen in Figure 1, a marked difference was not observed between the synonymous substitution rates of autosomes and X chromosome. This result indicates that the effect of "male-driven evolution" [25] is not strong. The male-driven evolution is a phenomenon in which the number of mutations is proportional to the number of germ-line cell divisions, so that the sperms have a higher rate of mutation than the eggs. There are large discrepancies among the results of previous studies with regard to the effect of male-driven evolution on mutation rates [26] In this study, a large variation in the rates of the substitution caused by CpG hypermutation among chromosomes and genes was observed. The mutation rate of the X chromosome was within the range of the mutation rate among autosomes, which is expected considering the variation in substitution rates among chromosomes. Thus, this finding suggests that the effect of male-driven evolution on the mutation rate was less than that of regional variation.

Vogel and Motulsky [27] pointed out that since the deamination of methyl-C occurs spontaneously and is independent of DNA replication, the rate of CpG mutations should be scaled with time and not with the number of cell divisions. Recently, Taylor et al. [14] investigated male mutation bias separately at non-CpG and CpG sites by using human-chimpanzee whole-genome alignments. They concluded that CpG hypermutation is weakly affected by the number of cell divisions. In this study, I demonstrated that the effect of male-driven evolution on CpG hypermutation is less strong than that of other chromosomal properties. Thus, although our result is similar to that of Vogel and Motulsky [27], I cannot exclude the findings of Taylor et al. [14]. Variation in the mutation rates among chromosomes must be one of the causes of the discrepancies among the results of previous studies with regard to the effect of male-driven evolution on mutation rates. Therefore, further study is warranted in this area.

Table 2 indicated that the CpG to TpG substitution rates were negatively correlated to the GC content of the chromosomes. This is consistent with the recent finding that the CpG mutation rates in the non-coding regions of human genes are positively correlated to the local GC content [11-14]. On one hand, table 2 showed that other types of substitution rates were positively correlated to the GC content of the chromosomes. On the other hand, multiple regression analyses shown in table 3 indicated that other types of substitution rates were negatively correlated to the GC content but the correlation was not significant. It should be noted that GC content, gene density, and frequency of CpG islands were correlated to each other. Discrepancy of these coefficients indicates that the effects of genetic features other than GC content on TpG to CpG substitution rates might have overcome that of GC content.

The results presented in this study also suggest that codon substitution rates are positively correlated to gene density. The reason of this correlation is unclear, but it might be due to fact that DNA melting is the rate-limiting step in cytosine deamination [28]. The difference between CpG hypermutability of the coding and non-coding regions may be due to the difference in the reason of DNA melting in the 2 regions. In the case of the coding regions, the cause of DNA melting is transcription; the DNA molecule separates into 2 single-strands before being transcribed. The frequency of transcription was positively correlated to the expression level of the gene, which in turn was positively correlated to the number of CpG islands [29]. In contrast, the reason for DNA melting in the non-coding region is not transcription but denaturation. The base pairs open one at a time, far below the melting temperature, and the half-life of individual GC base pairs are approximately 3 times longer than those of individual AT base pairs [30], so that the rate of CpG hypermutability in the non-coding region is positively correlated to the local GC content. If this is the case, CpG hypermutability will depend on the expression level of the genes. Recent study on mouse genes showed that the effect of gene expression level on codon bias is weaker than both the effect of gene expression level on amino acid composition and the effect of CpG hypermutability on codon bias [31], so that effect of gene expression level on mutation rate would be relatively small. Further study is needed in this area.

In this study, the codon substitution rates were estimated by using the coding regions of thousands of human and chimpanzee genes and inferring their ancestral sequences by assuming macaque genes as the outgroup. All types of substitutions show a significant negative correlation to Grantham's distance. This result indicates that the properties of amino acids are one of the major factors influencing the variation among non-synonymous substitutions, as previously reported [22].

The reason for the specific correlation found between codon substitution rates and chromosome sizes might be the physical distance between the genes and telomeres. Tyekucheva et al. [11] suggested that distances from telomeres play important roles on mutation rates. However, the numbers of substitutions are not very large; thus, dividing too many bins by the distance from the telomeres may yield weak results. As more data become available, incorporating these additional predictors in the regression analyses may be beneficial. Of special interest would be data on species other than humans.

On the basis of these results, a model that incorporates these genetic features was developed. The result shown above indicates that including GC content, gene density, CpG island density to the model improves the ability of prediction of the substitution rates

## Conclusion

The substitution rates of codons depend on gene densities of the chromosomes. Transcription-associated mutation is one such pressure. On the basis of these results, a model of codon substitutions that incorporates the effect of genomic features on codon substitution in human chromosomes was developed.

## Methods

### Data Set

To estimate the rates of codon substitutions, we used 10,372 orthologous gene trios obtained from human, chimpanzee, and macaque genomes [32]. The alignment of these genes comprised 4,717,227 codon triplets. Macaque genes were used as the outgroup. It was assumed that the human-chimpanzee divergence occurred 5 million years (MY) ago [33].

### Estimation of codon substitution rates by using the maximum parsimony (MP) method

I determined the codon sequences of the common ancestors of humans and chimpanzees by using the maximum parsimony (MP) method. Next, I counted the number of codon substitutions that had occurred along the human lineage. For some codon trios, the ancestral state between the human and chimpanzee codons appeared ambiguous when estimated by the MP method. In such cases, I treated all possible ancestral states equally. I also calculated the substitution rates by dividing the number of codon substitutions occurring annually by the number of ancestral codons. The awk program used in the analysis is available from the author upon request.

### Classification of codon substitutions

Because codon substitutions occur at different rates [22,24], they were classified into 4 categories: CpG to TpG substitutions, TpG to CpG substitutions, and non-CpG transitions and transversions. To distinguish CpG to TpG substitutions and TpG to CpG substitutions from non-CpG transitions, the adjacent nucleotides were also considered. For example, a codon substitution from CGC (Arg) to TGC (Ser) corresponds to a CpG to TpG substitution. A codon substitution from GGT, which codes Gly, to AGT, which codes Ser, corresponds to one CpG to TpG substitution if the third nucleotide of the 5'-adjacent codon is C. In contrast, a codon substitution from GGT to AGT is a non-CpG transition mutation if the third nucleotide of the 5'-adjacent codon is not C. If the observed nucleotide was T, its ancestral nucleotide was C, and the downstream nucleotide was G, the mutation was classified as a CpG type. If the observed nucleotide was A, its ancestral nucleotide was G, and the upstream nucleotide was C, the mutation was again classified as a CpG type. If the observed nucleotide was C, its ancestral nucleotide was T, and the downstream nucleotide was G, the mutation was classified as a TpG type. If the observed nucleotide was G, its ancestral nucleotide was A, and the upstream nucleotide was C, the mutation was again classified as a TpG type. Other types of substitutions are classified into two types: transitions and transversions [22,24,31].

### Evaluation of the chemical properties of amino acids

The frequency of amino acid substitutions relative to the frequency expected by chance decreases linearly with the increase in physicochemical differences between the amino acid pairs involved in a substitution. Grantham [34] proposed a formula for estimating the difference between amino acids; this formula combines the properties of amino acids such as composition, polarity, and molecular volume, which correlate best with the substitution frequencies of protein residues. In this study, I compared codon substitution rates with Grantham's [34] distance.

### Comparison between codon substitution rates and GC content, gene density and the density of CpG islands by using multiple regression analysis

I compared the codon substitution rates with the GC content and the gene and CpG island density, which are defined as the number of genes and CpG islands per MB of the chromosome, respectively. The GC content of each chromosome was calculated using the human reference sequence build 37. The gene density of each chromosome was calculated from the data in Ensembl release 57, and the density values of the CpG islands were taken from the study by Varriale and Bernardi [35]. Because of the lack of sufficient data, the relationship GC content, gene density and the density of CpG islands could not be analyzed. The substitution rates were then multiplied by 10^8 ^to simplify the calculation and transformed to their natural logarithms, as in a previous study [22]. These values were used in multiple regression analyses, which was performed using the statistical software R [36].

## Abbreviations

MP: maximum parsimony; BY: billion years; MY: million years; CpG: a cytosine followed by a guanine; MB: mega bases.

## Competing interests

The author declares that they have no competing interests.

## Authors' contributions

KM compared DNA sequences, conducted statistical analyses, and wrote the manuscript. KM read and approved the final manuscript.
